# Adeno-Associated Virus Mediated Gene Delivery: Implications for Scalable *in vitro* and *in vivo* Cardiac Optogenetic Models

**DOI:** 10.3389/fphys.2019.00168

**Published:** 2019-03-05

**Authors:** Christina M. Ambrosi, Gouri Sadananda, Julie L. Han, Emilia Entcheva

**Affiliations:** ^1^Department of Biomedical Engineering, Stony Brook University, Stony Brook, NY, United States; ^2^Department of Biomedical Engineering, George Washington University, Washington, DC, United States

**Keywords:** AAV, cardiac optogenetics, channelrhodopsin-2, LamR, sialic acid, iPS-CM, rat heart, gene therapy

## Abstract

Adeno-associated viruses (AAVs) provide advantages in long-term, cardiac-specific gene expression. However, AAV serotype specificity data is lacking in experimental models relevant to cardiac electrophysiology and cardiac optogenetics. We aimed to identify the optimal AAV serotype (1, 6, or 9) in pursuit of scalable rodent and human models using genetic modifications in cardiac electrophysiology and optogenetics, in particular, as well as to elucidate the mechanism of virus uptake. *In vitro* syncytia of primary neonatal rat ventricular cardiomyocytes (NRVMs) and human induced pluripotent stem cell-derived cardiomyocytes (hiPSC-CMs) were infected with AAVs 1, 6, and 9 containing the transgene for eGFP or channelrhodopsin-2 (ChR2) fused to mCherry. *In vivo* adult rats were intravenously injected with AAV1 and 9 containing ChR2-mCherry. Transgene expression profiles of rat and human cells *in vitro* revealed that AAV1 and 6 significantly outperformed AAV9. In contrast, systemic delivery of AAV9 in adult rat hearts yielded significantly higher levels of ChR2-mCherry expression and optogenetic responsiveness. We tracked the mechanism of virus uptake to purported receptor-mediators for AAV1/6 (cell surface sialic acid) and AAV9 (37/67 kDa laminin receptor, LamR). *In vitro* desialylation of NRVMs and hiPSC-CMs with neuraminidase (NM) significantly decreased AAV1,6-mediated gene expression, but interestingly, desialylation of hiPSC-CMs increased AAV9-mediated expression. In fact, only very high viral doses of AAV9-ChR2-mCherry, combined with NM treatment, yielded consistent optogenetic responsiveness in hiPSC-CMs. Differences between the *in vitro* and *in vivo* performance of AAV9 could be correlated to robust LamR expression in the intact heart (neonatal rat hearts as well as adult human and rat hearts), but no expression *in vitro* in cultured cells (primary rat cells and hiPS-CMs). The dynamic nature of LamR expression and its dependence on environmental factors was further corroborated in intact adult human ventricular tissue. The combined transgene expression and cell surface receptor data may explain the preferential efficiency of AAV1/6 *in vitro* and AAV9 *in vivo* for cardiac delivery and mechanistic knowledge of their action can help guide cardiac optogenetic efforts. More broadly, these findings are relevant to future efforts in gene therapy for cardiac electrophysiology abnormalities *in vivo* as well as for genetic modifications of cardiomyocytes by viral means *in vitro* applications such as disease modeling or high-throughput drug testing.

## Introduction

The use of adeno-associated viruses (AAVs) as transgene delivery vehicles in disease treatment requires comprehensive assessments of their performance and safety profiles. Advantages of AAVs include long-term expression, tissue tropism from 13 serotypes, and the ability to transduce both dividing and non-dividing cells (Aikawa et al., 2002; Muller et al., 2006; Williams et al., 2010; Srivastava, 2016). Recent clinical trials have explored the use of AAVs in the treatment of electromechanical consequences of heart failure, specifically in the upregulation of SERCA2a, a Ca^2+^ ATPase, known to be downregulated during the progression of the disease (CUPID; Jessup et al., 2011). Although a recent CUPID phase IIb trial concluded that the delivery of SERCA2a by AAV serotype 1 did not improve symptoms of heart failure in patients, no safety issues or adverse effects were observed (Greenberg et al., 2016). As of June 2017, there have been 183 clinical trials in humans using AAV^1^.

Concurrent to the exploration of AAV use in clinical trials, optogenetics has been rapidly developing as a promising tool in cardiac electrophysiology research (reviewed in Entcheva, 2013; Ambrosi et al., 2014; Montgomery et al., 2016; Pianca et al., 2017). Optogenetics relies on the genetic modification of cells and tissues to induce the expression of light-sensitive opsins for precise bi-directional control of activity. The technique allows for functional manipulation of target cells/tissues with high specificity through genetic modification, in addition to the superior spatiotemporal resolution afforded by optical means (Ambrosi et al., 2014). Consequently, the field of optogenetics requires highly efficient transgene delivery vehicles for cardiac applications. Such virally mediated optogenetic manipulations are “scalable” as they permit the parallel investigation of many cells *in vitro* for high-throughput all-optical cardiac electrophysiology (Dempsey et al., 2016; Klimas et al., 2016) and allow cardiac applications *in vivo* across different animal species, beyond the usual mouse transgenic models.

In this study, we investigated the efficiency and mechanisms of infection of three select AAV serotypes (1, 6, and 9) with known affinity for cardiac tissue in pursuit of scalable *in vitro* and *in vivo* models for cardiac optogenetics. Our study was motivated by the inconsistency of available data and study design evaluating serotype specificity in various animal models (see Supplementary Table S1 for a brief literature review). For instance, AAV9 has been shown to have highly efficient transgene delivery to the heart in the mouse and rat in a variety of studies (Inagaki et al., 2006; Pacak et al., 2006; Bish et al., 2008; Zincarelli et al., 2008); however, AAV1 and AAV6 are identified as superior for the heart in other studies (Kawamoto et al., 2005; Wang et al., 2005; Muller et al., 2006; Seiler et al., 2006; Palomeque et al., 2007; Zincarelli et al., 2008; Zhu et al., 2012; Kuken et al., 2015). In addition, developmental serotype specificity (i.e., preferential transgene expression in neonates versus adults) has also been suggested in studies involving dogs (Yue et al., 2008) and rhesus macaques (Pacak et al., 2006). A more recent work identified AAV6 as an efficient serotype for the infection of stem-cell derived cardiomyocytes (Rapti et al., 2015). Clinically and *in vivo*, AAV-mediated gene delivery is the approach of choice, including for expression of optogenetic tools. While a number of suitable options exist for gene delivery *in vitro* other than AAV-mediated gene transfer, there is often convenience in being able to utilize the same vectors for both studies *in vitro* and *in vivo*.

We used several experimental platforms relevant to the development of viral models for cardiac optogenetics. *In vitro* we assessed serotype performance in commonly used multicellular models of cardiac tissue – neonatal rat ventricular cardiomyocytes (NRVMs) and human induced pluripotent stem cell-derived cardiomyocytes (hiPSC-CM; Klimas et al., 2016). Adult rats were also systemically infected with AAVs as their larger size compared to mice allows for *in vivo* manipulations for cardiac research, including the insertion and implantation of fiber-based devices for long-term cardiac recording and stimulation (Klimas and Entcheva, 2014).

## Materials and Methods

Procedures involving animals were performed in accordance with institutional guidelines at both Stony Brook University (SBU) and George Washington (GW) University and conform to NIH guidelines for the care and use of laboratory animals. The reported experiments were prospectively approved by the GW Animal Care and Use Committee (IACUC) under numbers #A335 (for the neonatal rat culture) and #A339 (for the adult rat experiments).

Human heart tissue for protein analysis was procured through the Washington Regional Transplant Community (WRTC) program in Washington, DC, United States, and was provided to GW after de-identification by the procurement company.

Further details of the methods are provided in the Supplementary Material.

### In vitro

#### Cardiomyocyte Preparation

Neonatal rat ventricular cardiomyocytes were isolated using a previously published technique (Jia et al., 2011; Ambrosi et al., 2015). In short, cardiomyocytes from the ventricles of 2–3 day old Sprague–Dawley rats were enzymatically isolated with trypsin (USB, Cleveland, OH, United States) and collagenase (Worthington Biochemical Corporation, Lakewood, NJ, United States) and the presence of fibroblasts was minimized by pre-plating.

Frozen hiPSC-CMs (iCell Cardiomyocytes^2^; Cellular Dynamics, Madison, WI, United States) were thawed according to the manufacturer’s instructions. Cells were plated on fibronectin-coated (50 μg/mL; Fisher Scientific) glass-bottomed 96-well plates at a density of 156,000 cells/cm^2^.

#### Infection With AAV Serotypes and Ad-hChR2(H134R)-eYFP

Viral particles for pseudotyped AAV serotypes 1, 6, and 9 containing the transgene for eGFP were obtained from the University of Pennsylvania Vector Core (Philadelphia, PA, United States) or UPenn Core – AAV1/6/9.CB7.CI.eGFP.WPRE.rBG). The adenovirus (AdV) containing the transgene for channelrhodopsin2 fused to the reporter eYFP [Ad-CMV-hChR2(H134R)-eYFP] was prepared at the SBU Stem Cell Facility and characterized previously (Ambrosi and Entcheva, 2014).

Viral infection of NRVMs was completed in suspension immediately after cell isolation as described previously (Ambrosi and Entcheva, 2014). NRVMs were exposed to viral doses ranging in multiplicity of infection (MOI) from 100 to 2000 for AAV and 25 for AdV. Cells were plated on fibronectin-coated (50 μg/mL) glass-bottomed 96-well plates at a density of 400,000 cells/cm^2^.

hiPSC-CMs were infected after 5 days of culture once confluent monolayers had formed. Cells were exposed to viral doses ranging in MOI from 100 to 100,000 for AAV and 250 for AdV for a total of 2 h at 37°C.

#### Desialylation Treatment

To investigate the role of cell surface N-linked sialic acid in AAV infection, NRVMs and hiPSC-CMs were pre-treated with neuraminidase (NM; Type III, from *Vibrio cholera*; 25, 250, and 500 mU/mL; Sigma-Aldrich, St. Louis, MO, United States) for 2 h at 37°C prior to exposure to AAV particles as described above. NM, a broad-spectrum sialidase, has been shown to significantly reduce cell surface sialic acid and directly impact infectivity by AAVs 1 and 6 in a variety of other non-cardiac cell types (Wu et al., 2006).

#### TGF-β1 Treatment

To investigate the role of the 37/67 kDa laminin cell surface receptor in AAV9 infection, hiPSC-CMs were treated with recombinant human transforming growth factor-β1 (10 ng/mL; EMD Millipore) for 24 h at 37°C prior to infection. An existing report has shown upregulated LamR protein expression in cardiomyocytes upon TGF-β1 treatment (Wenzel et al., 2010).

#### Localization and Quantification of AAV Infection by eGFP

Monolayers were fixed with 3.7% formaldehyde 5 days after AAV infection. Cells were stained with DAPI (Fisher Scientific) and imaged using either an Olympus Fluoview FV1000 confocal system (for NRVMs) or a Nikon Eclipse TE2000U fluorescent system (for hiPSC-CMs) to quantify transgene (eGFP) expression.

#### Immunohistochemistry

Monolayers were permeabilized with 0.2% Triton-X 100 (Fisher Scientific) and stained with antibodies either for sarcomeric α-actinin (Sigma–Aldrich, St. Louis, MO, United States) or the 37/67 kDa laminin receptor (LamR) (Abcam, Cambridge, MA, United States). Secondary antibodies were conjugated to either AlexaFluor 488 or AlexaFluor 647 (Invitrogen).

#### Western Blots of LamR

Protein was extracted from adult human hearts available through the transplant program (ventricular portion of a middle-aged male and a middle-aged female patient’s hearts) and from human iPS-CMs (cultured for 7 days).

The antibody for the 37/67 kDa LamR receptor from Abcam was used in tandem with a fluorescent secondary antibody from Invitrogen to run the Western blots using protein collected from the cells and tissue samples. GAPDH antibody labeling (Abcam) was used as a normalization protein band, and ImageJ was used for quantification.

#### Optogenetic Control of the Engineered Cardiac Syncytium

Cell monolayers infected with Ad-CMV-hChR2(H134R)-eYFP (MOIs 25 and 250, respectively) or AAV9.CAG.hChR2(H134R)-mCherry.WPRE.SV40 (MOI 50,000–100,000 ± 500 mU/mL NM) were stained with the calcium- and voltage-sensitive dyes and optically mapped using our recently published all-optical, high-throughput system for dynamic cardiac electrophysiology, termed OptoDyCE (Klimas et al., 2016, 2018). The excitation filter for the actuating LED was 470/28 nm, the LED illumination for the voltage (di-4-ANBDQBS or Berst1) and calcium (Rhod-4AM) measurements was filtered as follows: 655/40 nm and 535/50, respectively. Fluorescence was collected by iXon Ultra 897 EMCCD; Andor, after passing through the emission filter 595/40 nm+700LP. Note that the UPenn Core considers the CAG and the CB7 promoters equivalent and uses them interchangeably; both are ubiquitous promoters, derivatives of CMV (Miyazaki et al., 1989).

### In vivo

#### Systemic Infection With AAV Serotypes

Adult male Sprague–Dawley rats (*n* = 4, 7–8 weeks old) were systemically injected with 0.5 × 10^12^ pseudotyped viral particles of serotypes 1 and 9 obtained from the UPenn Core – AAV1/9.CAG.hChR2(H134R)-mCherry.WPRE.SV40. The weight of the rats at the time of injection was between 220 and 250 g, therefore, the viral delivery was about 2.14 × 10^12^ vp/kg.

#### Localization and Quantification of AAV Infection by mCherry

Rats were anesthetized with a ketamine (75–95 mg/kg)/xylazine (5 mg/kg) cocktail and maintained on 1.5% isoflurane while a variety of tissues (including the heart, brain, liver, and kidney) were excised 4 weeks after viral injection. The tissues were fixed in 3.7% formaldehyde and imaged both macroscopically (IVIS Lumina Series III, PerkinElmer) and microscopically (Olympus Fluoview FV1000) for the presence of mCherry indicating successful transgene delivery.

#### Immunohistochemistry for LamR

Neonatal (2–3 days old, *n* = 2) and adult (11–12 weeks old, *n* = 2) rat hearts were fixed in 3.7% formaldehyde and embedded in paraffin. Tissue sections were stained with the polyclonal rabbit antibody for the 37/67 kDa LamR (Abcam, Cambridge, MA, United States) followed by a biotinylated anti-rabbit secondary antibody (Vector Laboratories, Burlingame, CA, United States). Breast carcinoma sections were used as a positive control.

#### Optogenetic Control of the Heart in the Open Chest

Functional assessment of transgene expression was tested by applying an epicardial S1 pacing protocol *in situ* in the open chest. Briefly, the rat was anesthetized with a ketamine (75–95 mg/kg)/xylazine (5 mg/kg) cocktail, intubated, and maintained on 1.5% isoflurane supplemented with oxygen throughout the procedure. The heart was exposed via a median sternotomy and optical stimulation was delivered with a fiber optics-coupled diode-pumped solid-state laser (470 nm; Shanghai Laser, Shanghai, China) directed on the left ventricular free wall. An ECG (Simple Scope, 2000; UFI, Morro Bay, CA, United States) was continuously recorded as the optical energy was increased in order to achieve 100% capture in the heart.

The heart, brain, liver, and kidney were excised from the animal and fixed in 3.7% formaldehyde. Fluorescent macroscopic and microscopic imaging for mCherry was then completed as described above.

### Statistics

All data are shown as the mean ± standard error of the mean (SEM). Statistically significant differences were identified using ANOVA followed by Tukey–Kramer’s test with a significance level of *p* < 0.05.

## Results

### AAV6 Outperforms AAV1 and AAV9 *in vitro*

**FIGURE 1:**
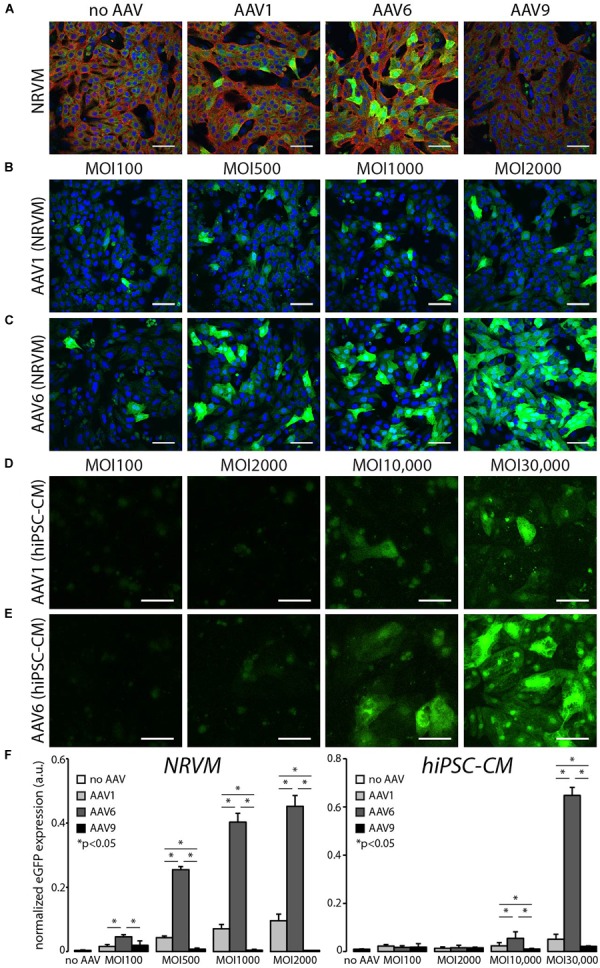
*In vitro* AAV6-mediated transgene expression is superior to the use of AAV1 and AAV9 in rat and human cardiomyocytes. **(A)** Cardiomyocyte-specific eGFP expression in NRVMs and hiPSC-CMs using AAV1, 6, and 9. AAV9-mediated expression did not exhibit levels of fluorescence above that of autofluorescence in non-infected control cells. Cell nuclei were labeled with DAPI (blue, NRVMS only), AAV-infected cells expressed eGFP (green), and cardiomyocytes were labeled with α-actinin (red); MOI 1000. **(B,D)** AAV1-mediated and **(C,E)** AAV6-mediated eGFP expression at four viral doses 5 days post-infection. hiPSC-CMs required viral doses two orders of magnitude greater than NRVMs (MOI 10,000 versus MOI 100) to show threshold eGFP expression. All scale bars are 50 μm and color-enhanced images are shown. **(F)** Quantification of the dose-dependent increase in eGFP expression in NRVMs and hiPSC-CMs. AAV6-mediated eGFP expression was significantly higher than AAV1-mediated expression at all viral doses. Data are presented as mean ± SEM (*n* = 3–7 independent samples per group). ^∗^Significance level at *p* < 0.05.

Recent applications of cardiac optogenetics *in vitro*, illustrating increased-throughput electrophysiology, require the use of viral vectors to deliver genetically encoded optical sensors or actuators (Leyton-Mange et al., 2014; Zhuge et al., 2014; Dempsey et al., 2016; Klimas et al., 2016). While typically AdV or lentiviral (LV) delivery has been employed in such applications, there is also interest in assessing the potential utilization of AAV vectors developed for *in vivo* applications as very few studies have been conducted in this area (Rapti et al., 2015). To systematically quantify serotype-specific and dose-dependent AAV infection, NRVMs and hiPSC-CMs were infected with AAVs 1, 6, and 9 containing the transgene for eGFP (Figure 1). Transgene expression was cardiomyocyte-specific, as eGFP was consistently co-localized in the same cells with positive immunostaining for α-actinin (Figure 1A). Although we employed strategies in the isolation of the NRVMs to reduce the presence of fibroblasts, a small number of fibroblasts are co-cultured with the cardiomyocytes, as can be seen in the non-eGFP/α-actinin-positive areas of Figure 1A. The hiPSC-CMs are, however, a purified population of cardiomyocytes and we have not observed any fibroblasts during culture.

AAV1- and AAV6-mediated eGFP expression was dose (MOI)-dependent in both NRVMs and hiPSC-CMs (Figure 1B–E). Quantification of the AAV-mediated dose-dependency of expression showed that infection by AAV6 resulted in significantly higher transgene expression at all MOIs for NRVMs and MOIs greater than 10,000 for hiPSC-CMs. Transgene expression due to AAV1 infection was also observed, but at significantly lower levels than AAV6-mediated expression. AAV9-mediated eGFP expression was not detected at these viral doses in either cell type (Figure 1F). It should also be noted that hiPSC-CMs require viral doses two orders of magnitude greater than NRVMs (MOI 10,000 versus 100) to show baseline eGFP expression.

Viral doses greater than those shown in Figure 1 resulted in significant cell death within the monolayers. Supplementary Figure S1 shows representative images of propidium iodide uptake (as a marker of dead cells) in NRVMs as a function of MOI. We quantified no significant differences in cell death across MOIs and serotypes; however, there is a trend toward increasing cell death with AAV1 infection at MOI 2000 (Supplementary Figure S1D).

### AAV9 Outperforms AAV1 *in vivo*

**FIGURE 2:**
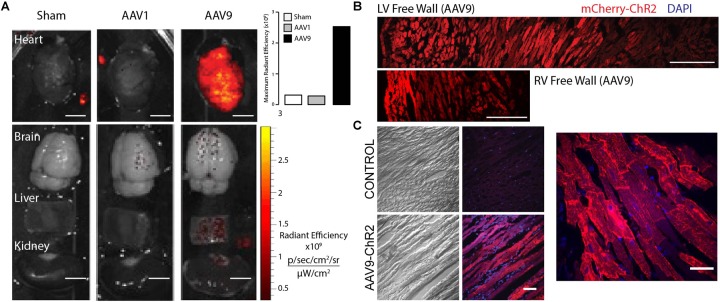
*In vivo* AAV9-mediated mCherry expression in the adult rat heart provides robust, predominantly cardiomyocyte-specific transgene delivery. **(A)** Systemic delivery of 0.5 × 10^12^ viral particles resulted in robust cardiac-specific expression of mCherry in 4 weeks using AAV9, but not AAV1 as measured using radiant efficiency. Other major organs (including brain, liver, and kidney) showed little to no signs of AAV-mediated infection. Scale bars are 500 μm. **(B)** AAV9-mediated transgene delivery resulted in transmural ChR2-mCherry expression in both the LV and RV free walls. Scale bars are 250 μm. **(C)** High-resolution images (brightfield and fluorescence) show cardiomyocyte-specific ChR2-mCherry expression using AAV9-mediated delivery. Scale bars are 50 μm.

A prior report on cardiac optogenetics, involving systemic delivery of AAV9 encoding for the channelrhodopsin-2 (ChR2) transgene, showed robust and long-lasting expression and functionality in mice (Vogt et al., 2015). However, except for a recent brief report (Nyns et al., 2016), to date this minimally invasive transduction approach has not been extended to larger animals, which may be more suitable for the study of cardiac arrhythmias due to size and ease of endoscopic access (Klimas and Entcheva, 2014). Here, systemic delivery of viral particles in the adult rat was employed through the lateral tail vein to assess the *in vivo* specificity of AAVs 1 and 9 (Figure 2). Unfortunately, the UPenn core does not offer AAV6 with ChR2, hence that serotype was not tested *in vivo* here. Four weeks after viral injection, excised hearts, brains, livers, and kidneys were assessed macroscopically for mCherry fluorescence (Figure 2A). AAV9-mediated infection resulted in global ventricular mCherry expression, while AAV1-mediated infection resulted in no cardiac transgene expression (with fluorescence comparable to sham viral injections) at a dose of 0.5 × 10^12^ viral particles per rat (equivalent to about 2.14 × 10^12^ vp/kg). Other excised organs (brain, liver, and kidney) showed little to no signs of AAV-mediated infection in all animals.

In rats infected with AAV9, mCherry expression in cardiomyocytes was robust and, not only expressed from apex to base as was observed with the macroscopic fluorescent imaging, but also expressed from the epicardium to the endocardium in both the left ventricular and right ventricular free walls (Figure 2B). Higher resolution microscopic imaging of AAV9-infected and sham hearts confirmed that observed fluorescence was not due to tissue auto-fluorescence and was localized to myocytes (Figure 2C).

### AAV Serotype Infection Is Mediated by Different Receptors on the Cardiomyocyte Surface

**FIGURE 3:**
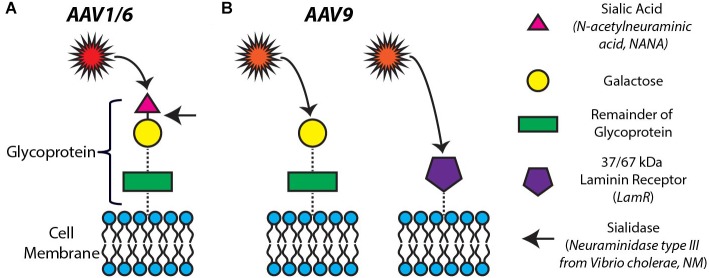
Proposed mechanisms of infectivity for AAV1, 6, and 9. **(A)** Cell surface N-linked sialic acid has been proposed as the primary receptor for AAV1 and 6 to infect and transduce cells. The removal of sialic acid by neuraminidase (targeting the portion of the glycoprotein indicated by the arrow) is expected to block the AAV1,6-mediated transduction of cells. **(B)** AAV9-mediated cell infection/transduction has been attributed to two receptors: terminal galactose on cell surface glycoproteins (left panel) and the 37/67 kDa laminin receptor (LamR) (right panel).

Previous studies have shown that infection by different AAV serotypes is mediated by a variety of cell surface receptors (for review, see Vance et al., 2015). Specifically, cell surface N-linked sialic acid has been proposed as the primary receptor for AAV1 and AAV6 to infect and transduce cells (Wu et al., 2006). There are at least two mechanisms of AAV9-mediated cell infection/transduction involving two different receptors: terminal galactose on cell surface glycoproteins (Shen et al., 2011) (that can be made available for AAV9 entry upon desialylation) and the 37/67 kDa LamR (Akache et al., 2006). Figure 3 provides a visual overview of the mechanisms of infectivity of cardiomyocytes we investigated in this study.

In order to probe the mechanisms of our differential observations of AAV serotype specificity *in vitro* (Figure 1) and *in vivo* (Figure 2), we explored the roles of both sialic acid and LamR in AAV-mediated transgene expression in cardiomyocytes. As indicated in Figure 3A by the arrow, we hypothesized that the removal of sialic acid by NM would block AAV1- and AAV6-mediated infection of cells. On the other hand, the same removal of sialic acid would also free up terminal galactose on the cell surface thus enhancing AAV9-mediated infection (Figure 3B, left panel). Similarly, AAV9 infection would be enhanced by the presence of LamR on the cell surface (Figure 3B, right panel).

### *In vitro* Desialylation Modulates AAV-Mediated Gene Expression

**FIGURE 4:**
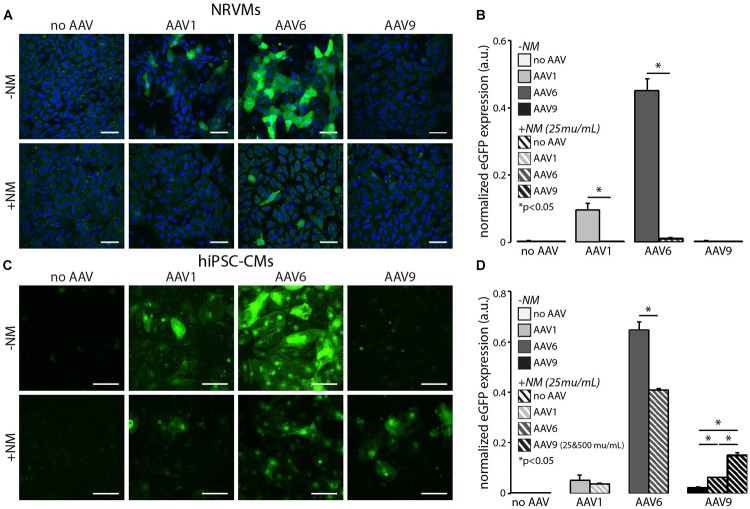
*In vitro* desialylation modulates AAV-mediated eGFP expression in NRVMs and hiPSC-CMs. Cardiomyocyte-specific eGFP expression with (+NM, 25 mU/mL) and without (−NM) neuraminidase treatment prior to viral infection in **(A)** NRVMs at MOI 2000 and **(C)** hiPSC-CMs at MOI 30,000. Cell nuclei were labeled with DAPI (blue, NRVMs only) and AAV-infected cells expressed eGFP (green). All scale bars are 50 μm and color-enhanced images are shown. Quantification of eGFP expression with and without desialylation in all three serotypes in **(B)** NRVMs and **(D)** hiPSC-CMs. eGFP expression mediated by AAV1 and 6 significantly decreased in both cell types, whereas transgene expression mediated by AAV9 significantly increased in hiPS-CMs only. Application of a higher dose of NM (500 mU/mL) in hiPSC-CMs infected with AAV9 resulted in even greater eGFP expression. Data are presented as mean ± SEM (*n* = 3–7 independent samples per group). ^∗^Significance level at *p* < 0.05.

Treatment of both NRVMs and hiPSC-CMs with NM, a broad spectrum sialidase, to remove cell surface sialic acid significantly reduced eGFP expression via AAV1 and AAV6 (Figure 4). In NRVMs, AAV1-mediated eGFP expression was completely abolished by 25 mU/mL NM, whereas AAV6-mediated expression was reduced to the point where only a few individual cells were eGFP-positive (Figure 4A,B). AAV9-mediated eGFP expression was unaffected in NRVMs as we did not observe the purported enhanced entry of AAV9 (Figure 3B) even at higher NM doses.

The same dose of NM in hiPSC-CMs never completely eliminated transgene expression, but AAV1 and AAV6-mediated infection was significantly reduced (Figure 4C,D), similar to the effect observed in NRVMs and in line with the predictions from Figure 3A. Interestingly, the application of NM to hiPSC-CMs in combination with AAV9 infection significantly increased transgene expression (Figure 4C,D). The additional application of 20× our standard NM dose (500 mU/mL), resulted in a further increase in AAV9-mediated gene expression, beyond that of AAV1-mediated expression without NM (Figure 4D), presumably by exposing terminal cell surface galactose for infection by AAV9, as illustrated in Figure 3B, and in contrast to our findings in NRVM.

### Expression of the 37/67 kDa Laminin Receptor (LamR) in the Intact Heart: Adult and Neonatal Rat Hearts and Adult Human Hearts

**FIGURE 5:**
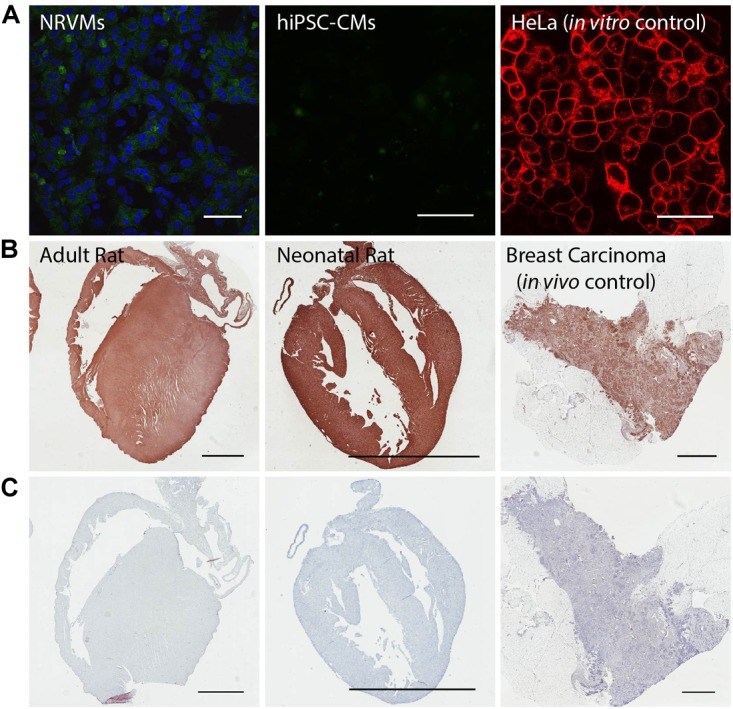
The 37/67 kDa LamR is expressed in the rat heart, but not in cultured NRVMs and hiPSC-CMs. **(A)** Negative *in vitro* immunostains of NRVMs and hiPSC-CMs for LamR. Concurrent immunostaining of HeLa cells, serving as a positive *in vitro* control for LamR. Scale bars are 50 μm. **(B)** Positive immunostains of adult and neonatal rat hearts. Concurrent immunostaining of breast carcinoma tissue, serving as a positive *in vivo* control for LamR. **(C)** Negative controls of tissue (stained with no primary LamR antibody) showed no contribution to the positive stain by non-specific secondary antibody staining. Scale bars in **B**,**C** are 3 mm.

The observed discrepancies in AAV serotype-mediated transgene expression *in vitro* (where AAV6 was most efficient) and *in vivo* (where AAV9 was most efficient) were further elucidated by investigating the presence of LamR which is purported to be a cell surface receptor for AAV9, as previously discussed. Our data show that LamR is not present *in vitro* in monolayers of NRVMs, nor in hiPSC-CMs (Figure 5A and Supplementary Figure S2A), but appears to be globally present *in vivo* in both the adult and neonatal rat heart (Figure 5B and Supplementary Figure S3B). Wild-type HeLa cells served as our *in vitro* positive control (Figure 5A) and tissue sections of breast carcinoma served as our *in vivo* positive control (Figure 5B and Supplementary Figure S3A). Experimental samples without the primary antibody showed that *in vivo* the secondary antibody did not yield any non-specific staining (Figure 5C).

**FIGURE 6:**
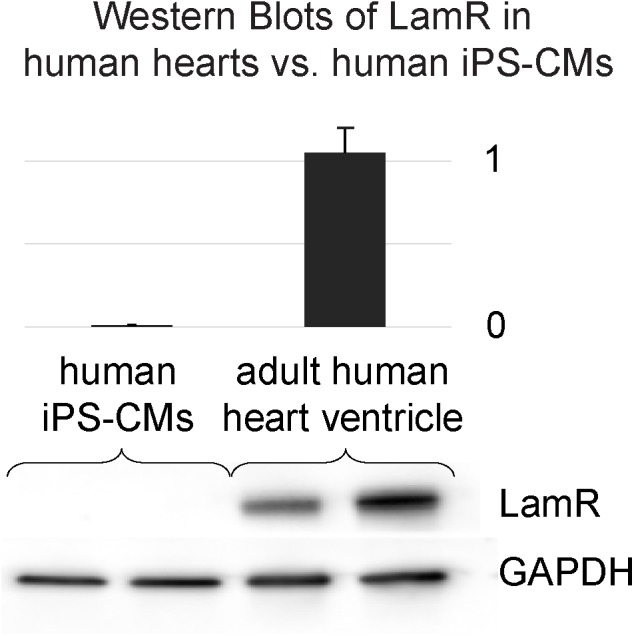
The 37/67 kDa LamR is expressed in the intact human heart. Western blots of LamR protein in fresh samples from male and female human hearts and from cultured hiPS-CMs corroborate the difference in expression (lack of LamR in the cultured myocytes and abundance in the intact human heart). GAPDH was used as a loading control and for normalization purposes.

These observations were extended to the adult human heart. Western blots of LamR protein in samples of fresh human hearts versus hiPS-CMs corroborated the difference in LamR expression between the intact heart and cardiomyocytes in culture (Figure 6).

Given our LamR expression data in NRVMs, hiPSC-CMs, rat and human hearts, it is important to note that the expression of LamR is dynamic and significantly affected by the tissue/culture environment. Specifically, we have observed the paucity of LamR expression in the *in vitro* environment with isolated cells (Figure 5A and Supplementary Figure S2) compared to its robust presence in intact tissues (Figure 5B, 6 and Supplementary Figure S3).

### TGF-β1 Treatment Does Not Significantly Affect AAV9-Mediated Gene Expression

Since the presence of LamR was not detected *in vitro* in NRVMs and hiPSC-CMs, we followed up on an earlier report (Wenzel et al., 2010) and pre-treated the monolayers with TGF-β1 (10 ng/mL for 24 h) in an attempt to increase LamR expression and facilitate AAV infectivity. Our data, however, show that TGF-β1 application does not significantly increase the expression of LamR (Supplementary Figures S2A,B) and minimally increases AAV9-mediated eGFP expression (Supplementary Figure S4).

### Viral Delivery of Optogenetic Tools

The growing use of optogenetics in cardiac applications motivated our search for optimized parameters for the optical control of the heart under various experimental conditions. One such application is the development of high-throughput all-optical electrophysiology for drug screening and cardiotoxicity testing (Dempsey et al., 2016; Klimas et al., 2016). The current study revealed that the environment (i.e., *in vitro* versus *in vivo*) is of great importance with regard to preferential serotype specificity. Cultured cardiomyocytes tend to lose cell surface receptors (LamR) critical to mediating *in vivo* AAV9 infection (Figure 5), although those receptors are present to some degree *in situ* in cultured explanted human cardiac tissue. Consequently, the specific environment may require different means for efficiently inscribing optical control.

**FIGURE 7:**
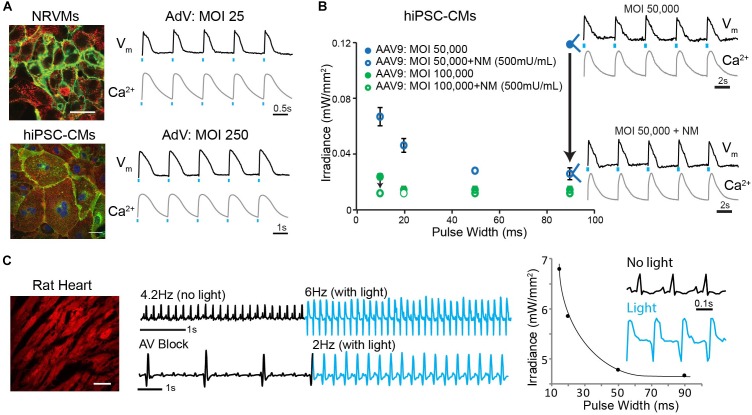
Robust *in vitro* and *in vivo* optogenetic control of the heart. **(A)** Adenoviral (AdV)-mediated ChR2-eYFP expression and functional measurements in NRVMs (MOI 25) and hiPSC-CMs (MOI 250) 2 days post-infection. Functional measurements were acquired using voltage- (di-4-ANBDQBS) and calcium- (Rhod4) sensitive dyes and example traces with optical pacing are shown. Cell nuclei were labeled with DAPI (blue) and AdV-infected cells expressed eYFP (green). Alpha-actinin staining (red) showed the cardiospecificity of the ChR2-eYFP infection. **(B)** Strength-duration curves for AAV9-mediated ChR2 expression in hiPSC-CMs. Conditions for infection included MOIs of 50,000–100,000 and NM applications of 500 mU/mL. Black arrows show the effect of NM treatment on lowering irradiance (mW/mm^2^) requirements; shown are voltage and calcium traces for the case of using MOI 50,000 without and with NM treatment. Data are presented as mean ± SEM (*n* = 3 per group). **(C)** AAV9-mediated ChR2-mCherry expression in the intact adult rat heart after 4 weeks results in optically sensitive myocardium *in situ* (left panel). A 0.8 mm diameter optical fiber was used to optically control electrical activity from the LV free wall as recorded using ECG (middle panel). Optical pacing resulted in an increased heart rate, as well as significant morphological changes in the QRS complex (right panel); the irradiance needed for this point stimulation was substantially higher than *in vitro*. Spatial scale bars are 50 μm in **B**,**C**. Temporal scale bars are as indicated.

AAV6-mediated transgene delivery resulted in acceptable expression levels, similar to those of our previous studies using AdV delivery (Ambrosi and Entcheva, 2014; Ambrosi et al., 2015; Klimas et al., 2016). However, the required dose (MOI) was orders of magnitude higher (Supplementary Figure S5), and the time required for transgene expression with AAV is not optimal for primary cells. Here AAV infection required 5 days for the cells to reach peak transgene expression, whereas in our previous studies >95% of NRVMs and hiPSC-CMs expressed ChR2 within 24–48 h using an AdV (Figure 7A; Ambrosi et al., 2015; Klimas et al., 2016). *In vitro* optical control was confirmed using all-optical electrophysiology (combining optical mapping by voltage- and calcium-sensitive dyes with simultaneous optogenetic stimulation; Klimas et al., 2016).

Although *in vitro* AAV9-mediated transgene delivery was deemed less optimal than AAV1,6-mediated delivery (Figure 1), successful expression of ChR2-mCherry using AAV9 was achieved under very specific conditions, as hypothesized and explored in this study (Figure 7B). A pre-treatment of hiPSC-CM monolayers with 500 mU/mL NM (20× the dose required to cause desialylation, Figure 4), followed by AAV9 infection at very high MOIs of 50,000–100,000 (5–10× the minimum dose for baseline transgene expression, Figure 1) resulted in optogenetic responsiveness. In all four cases (MOI 50,000 ± NM and MOI 100,000 ± NM), ChR2-mCherry was expressed resulting in an optically sensitive cardiac syncytium. However, at the lower concentration of MOI 50,000 only (no NM) and relevant low-light stimulation, only one out of three samples was optically excitable, and it only responded to long light pulses. As illustrated in Figure 7B, the strength-duration relationship showed the effect of NM treatment on improving optical responsiveness as compared to infection alone (black arrows). The strength-duration curve with NM treatment is similar to what we have reported previously with AdV in NRVM (for example, Yu et al., 2015). Despite successful transgene expression, infection at such high MOIs resulted in significant cell death (data not shown).

Extending this to the whole animal, here we show systemic delivery and successful expression of ChR2-mCherry in the adult rat heart 4 weeks after viral injection using AAV9 with a generic promoter (Figure 7C). Optical sensitivity was confirmed by rate and QRS morphology changes in the ECG, when using an optical fiber to deliver light to the left ventricle in the open chest of the anesthetized rat.

## Discussion

We investigated *in vitro* and *in vivo* AAV serotype specificity in rat and human models suitable as scalable experimental platforms for cardiac optogenetics. Different optimal serotypes were identified for *in vivo* and *in vitro* use. Namely, *in vitro* AAV6-mediated transgene expression was superior to AAV1,9-mediated delivery due to the presence of cell surface N-linked sialic acid (Figure 1, 4). The subsequent enzymatic removal of sialic acid significantly reduced or abolished AAV6- and AAV1-mediated gene delivery, independent of cell type. AAV1 and AAV6 are 99% homologous and belong to the same branch of the phylogenetic tree; the N-linked sialic acid receptor has been suggested as the primary receptor for both of these serotypes (as also corroborated by our data), yet it is recognized that the AAV viral entry is more complex (Vance et al., 2005). For example, the secondary receptor for AAV6 is reported to be the epidermal growth factor receptor (EGFR), while for AAV1 the secondary receptor remains unknown. These differences can explain the quantitatively different performance of the two serotypes in our cells despite qualitatively similar response.

AAV9, on the other hand, belongs to a separate branch on the phylogenetic tree and shares 82% homology with AAV1, 6 and with the most widely used in clinical trials AAV2(Vance et al., 2005). Interestingly, the same desialylation process that suppressed AAV6 and AAV1 entry enhanced AAV9-mediated expression but only in hiPSC-CMs (Figure 4). In contrast, *in vivo* serotype specificity in the adult rat favored delivery by AAV9, likely mediated by the presence of cell surface LamR (Figure 2, 5). The latter appears ubiquitous in the intact heart but could not be found in cultured cardiomyocytes (absence confirmed in primary neonatal rat myocytes and in human iPS-CMs). An argument against cardiomyocyte maturity being the central driver for LamR expression or its loss is presented by the observation that the intact neonatal rat heart, with presumably less mature cells, has just as strong expression of LamR as the adult rat heart (Figure 5B). In this study, fibronectin-coated dishes were used only. The composition of the extracellular matrix may affect viral uptake – for example, for cancer cells expressing LamR (e.g., HeLa), addition of laminin decreased the expression of the receptor and the viral uptake (Akache et al., 2006). Other culture-related conditions, including proper mass transport, oxygenation, proper fuel/nutrients in the medium, may influence the LamR expression.

The emergence of human stem-cell derived cardiomyocytes and their combination with genetically encoded sensors and actuators (Leyton-Mange et al., 2014; Zhuge et al., 2014; Dempsey et al., 2016; Klimas et al., 2016) has prompted a closer look at the performance of various viral vectors, including AAVs (Rapti et al., 2015) due to the convenience of sharing the usage of such vectors for both *in vivo* and *in vitro* applications. The results presented here, showing preferential infectivity of cardiomyocytes *in vitro* (AAV6 > AAV1 >> AAV9), are consistent with a recent report in human stem-cell derived cardiomyocytes (Rapti et al., 2015). Interestingly, we find that the ease of viral infection in the *in vitro* environment seems to be dependent on two major factors: the viral vector itself (AAV, AdV, or lentivirus) and the state of differentiation of the target cell (Supplementary Figure S5). In our experience, primary cardiomyocytes are the easiest to infect (i.e., requiring the lowest viral doses for >80% cell transgene expression) using AdV (Ambrosi and Entcheva, 2014; Ambrosi et al., 2015) and AAV (explored in this study). iPSC-CMs require 10–100× increased viral doses compared to primary cardiomyocytes for the same efficiency of expression, and the presumably least differentiated cells, cardiac fibroblasts, require the highest viral doses (Yu and Entcheva, 2016), although we have not tested AAVs on the latter cell type (Supplementary Figure S5). Similar observations have been reported for pluripotent stem cells before and after differentiation into cardiomyocytes (Rapti et al., 2015). While AAV delivery appears sub-optimal for *in vitro* use (compared to LV or AdV application), our dissection of the mechanism of viral entry suggests some strategies to improve infectivity with select AAVs, e.g., desialylation enhances AAV9-mediated entry, while sialic acid on the cardiomyocyte surface promotes AAV6 entry.

Optogenetics in the intact organism requires the genetic modification of cells and tissues, and hence it necessitates the development of efficient, safe tools for gene therapy. Methods for non-viral transfer of genetic material, including electroporation, ballistic DNA transfer, and cationic lipid-based gene transfer, are known to be less efficient and the persistence of transgene expression is short-lived (Ramamoorth and Narvekar, 2015). Therefore, viral transfer of genetic material through the use of AdVs, lentiviruses, and AAVs is desirable. AAVs are preferred due to their comparatively low immunogenicity (Zaiss et al., 2002). *In vivo*, AAV-mediated transgene delivery has been used for cardiac optogenetics in rodent hearts (Nussinovitch and Gepstein, 2015; Vogt et al., 2015; Nyns et al., 2016). AAV9-mediated expression of ChR2 in the mouse heart yielded highly efficient and cardiac-specific transduction when applied by a minimally invasive systemic route (Vogt et al., 2015); a recent report used a similar delivery but with a very high dose of cardiac-specific viral vector in the rat (Nyns et al., 2016). Direct cardiac injections of AAV9 encoding for the ChR2 transgene also resulted in optical responsiveness of the rat heart (Nussinovitch and Gepstein, 2015). However, systemic delivery is preferred not only because of its minimally invasive nature (and hence, suitability for translation), but also because of better uniformity of expression (Prasad et al., 2011; Vogt et al., 2015).

The purpose of this study was to provide practical information to users of commercially available viral constructs as much as possible to ensure easy reproducibility. Here, we used only commercially available constructs for the tested AAV viruses, obtainable through the UPenn Core, and we stayed consistent when comparing the different serotypes. No commercial version was available for AAV6 with ChR2 through the UPenn Core; therefore, it was not included in the *in vivo* tests. For the AdV studies *in vitro*, we have developed viral vectors, and these are available to outside investigators upon request. Different promoters were used for different portions of this study, limited by the commercially available viral vectors. All serotypes of AAV (1,6,9) used either CAG or CB7 promoter, while the AdV constructs had the CMV promoter. CMV, CAG, and CB7 are all strong ubiquitous promoters that are commonly used. CAG/CB7 are considered identical and interchangeable by the UPenn Core and there is no literature to differentiate between the performance of the two. CAG/CB7 is a synthetic promoter, a derivative of CMV with added transcribed sequence from chicken beta-actin gene and enhancer elements (Miyazaki et al., 1989). In most cases, CAG/CB7 is considered a stronger version of CMV.

The direct comparisons between AAV serotypes were done using identical promoters to avoid influence by this factor. For example, the revealed superior performance of AAV6 *in vitro* compared to AAV1 and 9 is not impacted by the promoter itself. Similarly, the superior performance of AAV9 over AAV1 for *in vivo* optogenetics is not influenced by the promoter itself, CAG/CB7. Considering the more potent CAG/CB7, compared to the CMV used with the AdV vectors *in vitro*, the dramatically better performance of AdV delivery over AAV serotypes *in vitro* also holds true.

AAV serotypes 1, 6, and 9 have shown different degrees of gene transfer to the heart (Supplementary Table S1). The specificity of cardiac transduction is dose-dependent. For example, AAV9 delivered systemically in mice at 10^11^ MOI is rather cardiac-specific without affecting other organs; however, at 10^12^ MOI, it also transduces liver, skeletal muscle, and pancreas (Inagaki et al., 2006). Scaling of viral dose from mice to rats by body weight ratio yields about 5–10 times higher amount of virus needed for cardiac-specific transduction. Indeed, 10^12^ MOI in rats showed heart-specific transduction with AAV9 (no expression in liver, kidney, brain, lung; Cataliotti et al., 2011). Our results are similar. In addition to the AAV tissue tropism, the use of cardiac-specific promoters, such as cardiac troponin T (cTnT), has been shown to further increase specificity; however, the level of expression derived from tissue-restricted promoters may not be as high as from ubiquitous viral promoters (Prasad et al., 2011).

Of critical importance in the use of AAV serotypes for optimized gene therapy applications is consideration of the mechanism of infectivity. In this study, we not only identified optimal serotypes for *in vitro* and *in vivo* use, but also explored the mechanism of infection and fundamental differences between experimental platforms. Specifically, our results are consistent with AAV9 infection being mediated by either terminal galactose (Figure 4, only in hiPS-CMs) or LamR (Figure 5). Identification of unique cell surface receptors in the heart and other organs will continue to drive the design of truly optimized AAV serotypes for cardiac electrophysiology applications, such as optogenetics, and beyond.

## Data Availability

The raw data supporting the conclusions of this manuscript will be made available by the authors, without undue reservation, to any qualified researcher.

## Author Contributions

CA, GS, and JH performed the experiments, analyzed the data, and produced figures. CA conducted the rat experiments. GS and CA performed the *in vitro* experiments. JH analyzed protein expression. EE and CA conceived the study and oversaw the project. CA and GS produced an initial draft of the manuscript, which was edited by EE.

## Conflict of Interest Statement

The authors declare that the research was conducted in the absence of any commercial or financial relationships that could be construed as a potential conflict of interest.

## Footnotes

1 www.abedia.com/wiley
