# Supplementary material for: Adeno-Associated Virus Mediated Gene Delivery: Implications for Scalable in vitro and in vivo Cardiac Optogenetic Models
Source: Front Physiol. 2019 Mar 5;10:168. doi: 10.3389/fphys.2019.00168 (PMC6412093; doi:10.3389/fphys.2019.00168)
Supplement: Supplementary file 1 [file Data_Sheet_1.pdf]

Supplementary Material

Adeno-associated virus mediated gene delivery: Implications for scalable *in vitro* and *in vivo* cardiac optogenetic models

Christina M. Ambrosi, Ph.D.[*], Gouri Sadananda, B.S., Julie L. Han, B.S., and Emilia Entcheva, Ph.D.

*** Correspondence:** Emilia Entcheva, Ph.D.: entcheva@gwu.edu

1 Supplementary Methods

Procedures involving animals were performed in accordance with institutional guidelines at both Stony Brook University and George Washington University and conform to NIH guidelines for the care and use of laboratory animals. Procedures involving human hearts were approved by the George Washington University Institutional Review Board and conform to federal regulations.

1.1 *In Vitro*

1.1.1 Cardiomyocyte Preparation

Neonatal rat ventricular cardiomyocytes (NRVM) were isolated using a previously published technique.(Jia et al., 2011; Ambrosi et al., 2015) In short, cardiomyocytes from the ventricles of 2-3 day old Sprague-Dawley rats were enzymatically isolated with trypsin (1 mg/mL; USB, Cleveland, OH) and collagenase (1 mg/mL; Worthington Biochemical Corporation, Lakewood, NJ). The majority of fibroblasts were removed by a two-step pre-plating procedure. NRVMs were then cultured in M199 media (Invitrogen, Grand Island, NY) supplemented with 12µM L-glutamine (Invitrogen), 0.05 µg/mL penicillin-streptomycin (Mediatech Cellgro, Kansas City, MO), 0.2 µg/mL vitamin B12 (Sigma-Aldrich, St. Louis, MO), 10mM HEPES (Invitrogen), 3.5 mg/mL D-(+)-glucose (Sigma-Aldrich), and 10% fetal bovine serum (FBS; Invitrogen).

Frozen human induced pluripotent stem cell-derived cardiomyocytes (hiPSC-CMs, iCell Cardiomyocytes[2]; Cellular Dynamics, Madison, WI) were thawed according to the manufacturer's instructions. Cells were plated on fibronectin-coated (50 µg/mL; Fisher Scientific) glass-bottomed 96 well plates (Cellvis, Mountain View, CA) at a density of 156,000 cells/cm^2. Media was changed every other day throughout culture. Within four days the monolayers were ready for experiments.

1.1.2 Infection with AAV Serotypes and Ad-hChR2(H134R)-eYFP

Viral particles for pseudotyped AAV serotypes 1, 6, and 9 containing the transgene for eGFP were obtained from the University of Pennsylvania Vector Core (Philadelphia, PA; AAV1/6/9.CB7.CI.eGFP.WPRE.rBG). The adenovirus (AdV) containing the transgene for channelrhodopsin2 fused to the reporter eYFP (Ad-hChR2(H134R)-eYFP) was prepared at the Stony Brook University Stem Cell Facility and characterized previously.(Ambrosi and Entcheva, 2014;

Ambrosi et al., 2015) All viruses tested in this study contained ubiquitous promoters (CAG/CB7 for AAVs and CMV for AdV).

Viral infection of NRVMs was completed in suspension immediately after cell isolation as described previously.(Ambrosi and Entcheva, 2014) NRVMs were exposed to viral doses ranging in multiplicity of infection (MOI) from 100 to 2,000 for AAV and 25 for AdV. During the infection, cells were temporarily suspended in lower serum (2%) M199 media at 37ºC and manually agitated every 15-20 minutes for a total of two hours. After the infection was completed, NRVMs were plated on fibronectin-coated (50 µg/mL; Fisher Scientific) glass-bottomed 96 well plates (Cellvis, Mountain View, CA) at a density of 400,000 cells/cm^2.

hiPSC-CMs were infected after five days of culture once confluent monolayers had formed. Cells were exposed to viral doses ranging in MOI from 100 to 100,000 for AAV and 250 for AdV for a total of two hours at 37ºC. Similar to the NRVM infection, hiPSC-CMs were also manually agitated every 15-20 min during incubation.

1.1.3 Desialylation Treatment

To investigate the role of cell surface N-linked sialic acid in AAV infection, NRVMs and hiPSC-CMs were pre-treated with neuraminidase (Type III, from *Vibrio cholera*; 25, 250, and 500 mU/mL; Sigma-Aldrich, St. Louis, MO) for two hours at 37ºC prior to exposure to AAV particles as described above. Neuraminidase, a broad-spectrum sialidase, has been shown to significantly reduce cell surface sialic acid and directly impact infectivity by AAVs 1 and 6 in a variety of other non-cardiac cell types.(Wu et al., 2006)

1.1.4 TGF-β1 Treatment

To investigate the role of the 37/67 kDa laminin cell surface receptor in AAV9 infection, hiPSC-CMs were treated with recombinant human transforming growth factor-β1 (10ng/mL; EMD Millipore) for 24 hours at 37ºC prior to infection. An existing report has shown upregulated LamR protein expression in cardiomyocytes upon TGF-β1 treatment.(Wenzel et al., 2010)

1.1.5 Localization and Quantification of AAV Infection by eGFP

Monolayers were fixed with 3.7% formaldehyde five days after AAV infection. Cells were stained with DAPI (Fisher Scientific) and imaged using either an Olympus Fluoview FV1000 confocal system (for NRVMs) or a Nikon Eclipse TE2000U fluorescent system (for hiPS-CMs). Fluorescent images were binarized with thresholds determined by ensuring that autofluorescence on control cells not infected with AAV were not detectable as eGFP expression. Those binarization thresholds were then applied to cells infected with AAV and expressing eGFP. eGFP expression was calculated as the percent of pixels above threshold and normalized to control samples. Percent expression values based on pixel counts (as shown in **Figures 1** and **4**) should not be interpreted as percent infected cells since they report lower values than actually seen, as non-cellular/non-myocyte space was not corrected for.

1.1.6 Immunohistochemistry

After fixation and image acquisition for eGFP quantification, select NRVM monolayers were permeabilized with 0.2% Triton-X 100 (Fisher Scientific) and stained with monoclonal mouse

antibodies either for sarcomeric α-actinin (Sigma-Aldrich, St. Louis, MO) or the 37/67 kDa laminin receptor, LamR (Abcam, Cambridge, MA). Select hiPSC-CM monolayers were stained with a rabbit monoclonal antibody the 37/67 kDa laminin receptor (ab133645, Abcam, Cambridge, MA). The secondary antibody was either a goat anti-mouse secondary conjugated to Alexa Fluor 647 or a goat anti-rabbit antibody conjugated to Alexa Fluor 488 (Invitrogen). Wild-type HeLa cells were used as a positive LamR control.

1.1.7 Western Blots of LamR

Protein was extracted from fresh adult human hearts available through the transplant program (ventricular portion of a middle-aged male and a middle-aged female patient's hearts) and from human iPS-CMs (cultured for 7 days, 1.5mil cells per sample). Whole cell and tissue samples were extracted with Qproteome Mammalian Protein Prep Kit (Qiagen) according to manufacturer's protocol, fractionated by SDS-PAGE, and transferred to nitrocellulose membrane using Trans-Blot Turbo Transfer System according to the manufacturer's protocols (Bio-Rad).

The antibody for the 37/67 kDa LamR receptor from Abcam was used in tandem with a chemiluminescent HRP secondary antibody from Abcam (ab6721) to run the Western blots using protein collected from the cells and tissue samples. GAPDH antibody labeling (ab181602, Abcam) was used as a normalization protein band, and ImageJ was used for quantification.

After blocking with 5% nonfat milk in TBST, the membrane was washed three times with TBST and incubated with the primary antibodies against LamR (1:200) and GAPDH (1:800) at 4°C for 12 h. Membranes were washed three times for 5 min and incubated with a 1:2000 dilution of horseradish peroxidase-conjugated or anti-rabbit antibodies for 2 h. Blots were washed with TBST three times and developed with the ECL system (ThermoFisher Scientific) according to the manufacturer's protocols. ImageJ was used to quantify the LamR-to-GAPDH ratios for each sample.

1.1.8 Optogenetic Control of the Engineered Cardiac Syncytium

Cell monolayers (comprised of either NRVMs or hiPSC-CMs) globally infected with AdV-hChR2(H134R)-eYFP (MOIs 25 and 250, respectively) or AAV9.CAG.hChR2(H134R)-mCherry.WPRE.SV40 (MOI 50,000-100,000 ± 500mU/mL NM) were stained with the calcium-sensitive dye Quest Rhod4 AM (10µM; AAT Bioquest, Sunnyvale, CA) and the voltage-sensitive dye di-4-ANBDQBS (35µM; from Dr. Leslie Loew, University of Connecticut) and optically mapped using our recently-published all-optical, high-throughput system for dynamic cardiac electrophysiology, termed OptoDyCE.(Klimas et al., 2016; Klimas et al., 2018). Illumination for optical actuation and sensing was delivered by TTL-programmable LEDs coupled to the OptoDyCE platform. Strength-duration curves were constructed using pulse widths ranging from 10 to 90 ms. The excitation filter for the actuating LED was 470/28 nm, the LED illumination for the voltage (di-4-ANBDQBS or Berst1) and calcium (Rhod-4AM) measurements was filtered as follows: 655/40 nm and 535/50 respectively. Fluorescence was collected by iXon Ultra 897 EMCCD; Andor, after passing through the emission filter 595/40 nm+700LP. Note that the UPenn Core considers the CAG and the CB7 promoters equivalent and uses them interchangeably; both are ubiquitous promoters, derivatives of CMV (Miyazaki et al., 1989).

1.2 *In Vivo*

1.2.1 Systemic Infection with AAV Serotypes

Adult male Sprague-Dawley rats (n=4, 7-8 weeks old) were systemically injected with $0.5x10^{12}$ pseudotyped viral particles of serotypes 1 and 9 obtained from the University of Pennsylvania Vector Core (Philadelphia, PA; AAV1/9.CAG.hChR2(H134R)-mCherry.WPRE.SV40). Viral particles were diluted in 400µL sterile saline and injected through the lateral tail vein with a 26-gauge needle. Additional sterile saline (400µL) was then flushed through the needle to ensure that all of the viral particles were injected into the animal. Sham rats (n=2) were injected with corresponding amounts of sterile saline only. All rats were monitored daily and weighed biweekly.

1.2.2 Localization and Quantification of AAV Infection by mCherry

Rats were anesthetized with a ketamine (75-95mg/kg)/ xylazine (5mg/kg) cocktail and maintained on 1.5% isoflurane while a variety of tissues (including the heart, brain, liver, and kidney) were excised four weeks after viral injection. The tissues were fixed in 3.7% formaldehyde and imaged both macroscopically and microscopically for the presence of mCherry indicating successful transgene delivery. Macroscopic fluorescence imaging was completed using an IVIS Lumina Series III imaging system (PerkinElmer). Macroscopic mCherry fluorescence was quantified by radiant efficiency. Tissues were then embedded and frozen in Tissue-Tek® OCT Compound (Electron Microscopy Sciences, Hatfield, PA) and stored at -80ºC. The tissues were cryosectioned at 16 µm and mounted on Superfrost Plus glass slides (Fisher Scientific). High resolution microscopic images of cell-specific mCherry expression were then acquired using an Olympus Fluoview FV1000 confocal imaging system.

1.2.3 Immunohistochemistry for LamR

Neonatal (2-3 days old, n=2) and adult (11-12 weeks old, n=2) rat hearts were fixed in 3.7% formaldehyde and embedded in paraffin. Tissue sections were stained with the polyclonal rabbit antibody for the 37/67 kDa laminin receptor (Abcam, Cambridge, MA) followed by a biotinylated anti-rabbit secondary antibody (Vector Laboratories, Burlingame, CA). Concurrent hematoxylin staining was also performed to identify the full tissue sections. Breast carcinoma sections were used as a positive control.

1.2.4 Optogenetic Control of the Heart in the Open Chest

Functional assessment of transgene expression was tested by applying an epicardial S1 pacing protocol *in situ* in the open chest. Briefly, the rat was anesthetized with a ketamine (75-95 mg/kg)/ xylazine (5 mg/kg) cocktail, intubated, and maintained on 1.5% isoflurane supplemented with oxygen throughout the procedure. The heart was exposed via a median sternotomy and optical stimulation was delivered with a fiber optics-coupled diode-pumped solid state laser (470nm; Shanghai Laser, Shanghai, China) directed on the left ventricular free wall. An ECG (Simple Scope 2000; UFI, Morro Bay, CA) was continuously recorded as the optical energy was increased in order to achieve 100% capture in the heart.

The heart, brain, liver, and kidney were excised from the animal and fixed in 3.7% formaldehyde. Fluorescent macroscopic and microscopic imaging for mCherry was then completed as described above.

1.3 Statistics

All data are shown as the mean ± standard error of the mean (S.E.M.). Statistically significant differences were identified using ANOVA followed by Tukey-Kramer's test with a significance level of $p<0.05$.

Table S1. Brief Literature Review of Cardiac AAV Serotype Studies

Study Authors	Animal Model	Serotypes	Promoter[a]	Route of Administration[b]	Time Course
Aikawa, et al.(Aikawa et al., 2002)	mouse	2	CMV, MHC	direct cardiac injection	16 weeks
Wang, et al.(Wang et al., 2005)	mouse, hamster	1,2,5,6,7,8	CB, CMV	IP/IV	5 months
Kawamoto, et al.(Kawamoto et al., 2005)	rat	2,6	CAG	IM	1 week
Inagaki, et al.(Inagaki et al., 2006)	mouse	8,9	E1α, CMV	IP, SC	10-13 days
Pacak, et al.(Pacak et al., 2006)	mouse	1,8,9	CMV	IV	56 days
Seiler, et al.(Seiler et al., 2006)	mouse	5,6	RSV	nasal aspiration	1-6 months
Muller, et al.(Muller et al., 2006)	mouse, rat	2,6	CMV	IV, direct cardiac injection	3 weeks, 12 months
Palomeque, et al.(Palomeque et al., 2007)	rat	1,2,3,4,5,6,7,8	CMV	direct cardiac injection	24 weeks
Bish, et al.(Bish et al., 2008)	mouse, rat	1,6,7,8,9	CMV	IP, direct cardiac injection	1 month, 1 year
Kuken et al. (Kuken et al., 2015)	rabbit	1	CMV	IP	4 weeks
Zhu et al. (Zhu et al., 2012)	dog	6	CMV	IM	12 weeks
Zincarelli, et al.(Zincarelli et al., 2008)	mouse	1,2,3,4,5,6,7,8,9	CMV	IV	9 months
Yue, et al.(Yue et al., 2008)	dog	9	RSV	IM, IV	2-10 weeks, 6 months
Yang, et al.(Yang et al., 2009)	mouse, hamster	1,6,7,8,9	CB, CMV	IV	2 weeks
Ying, et al.(Ying et al., 2010)	mouse	2,9	CMV	IV	1 month
Prasad, et al.(Prasad et al., 2011)	mouse	1,2,6,8,9	cTnT	IV	4 weeks
Geisler, et al.(Geisler et al., 2011)	mouse	9	CMV	IV	4 weeks
Pulicherla, et al.(Pulicherla et al., 2011)	mouse	9	CAG	IV	1-4 weeks

[a]*Promoter Abbreviations*
CMV – cytomegalovirus
MHC – myosin heavy chain
CB/CAG – CMV enhancer/ chicken beta-actin
E1α – elongation factor-1 alpha
RSV – Rous sarcoma virus
cTnT – cardiac troponin T

[b]*Route of Administration Abbreviations*
IP – intraperitoneal
IV – intravenous
IM – intramuscular
SC – subcutaneous

Supplementary Figures

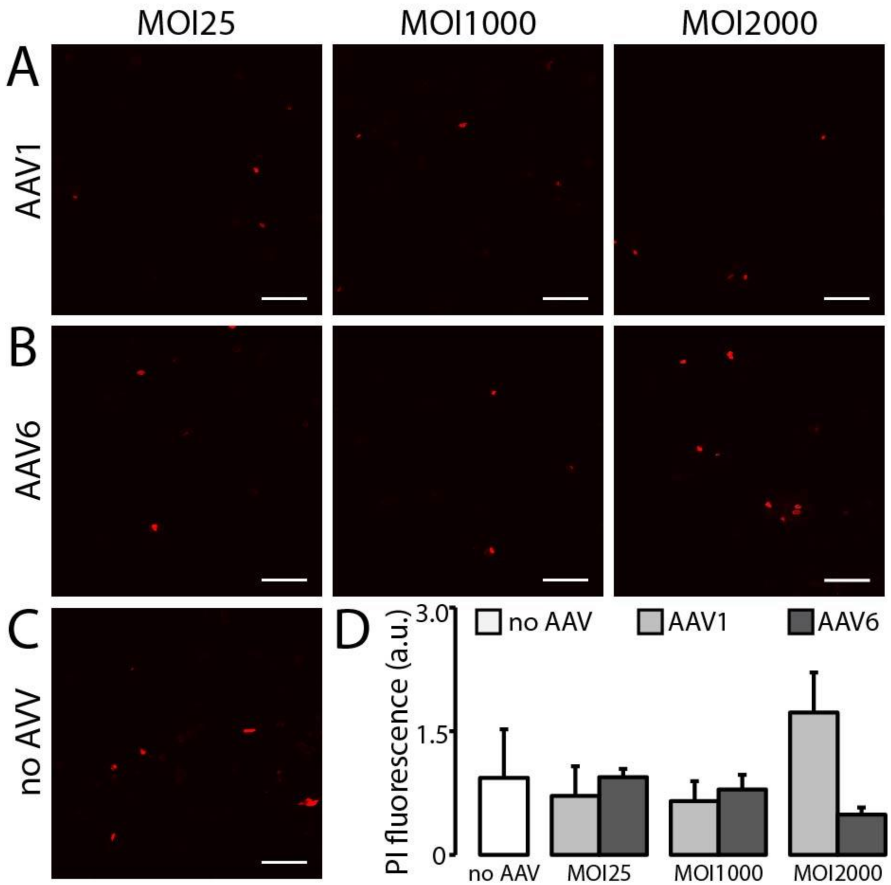

Supplementary Figure 1. AAV-mediated infection causes no significant cell death in NRVMs. The uptake of propidium iodide (PI, red; representative of dead cells) in (A) AAV1-infected cells, (B) AAV6-infected cells, and (C) non-infected control cells. The viability of infected cells was assessed at MOIs 25, 1000, and 2000. Scale bars are 50µm. (D) Quantification of cell death shows no significant differences in viability across groups. Data are presented as mean±S.E.M. (n=2-3 per group).

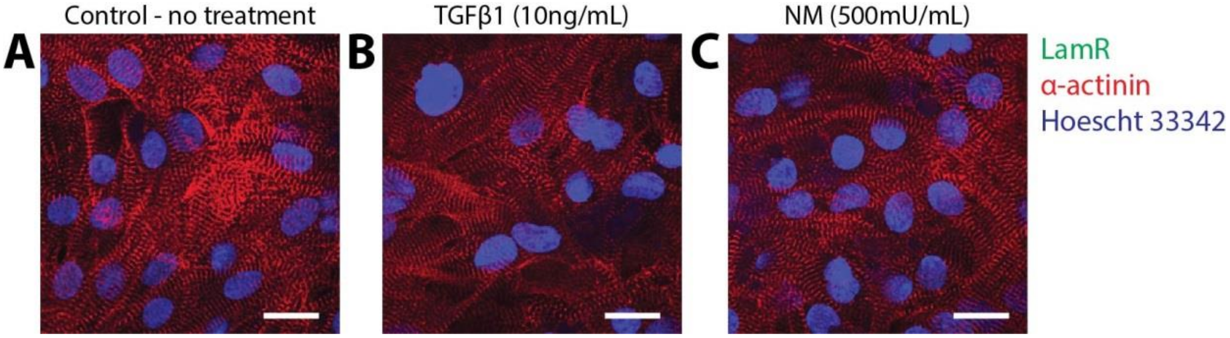

Supplementary Figure 2. LamR is not expressed *in vitro* in hiPSC-CMs. (**A**) Control cells, (**B**) cells treated for 24 hours with TGFβ1 (10ng/mL), and (**C**) cells treated for two hours with NM (500mU/mL) did not stain for LamR. Cell nuclei were labeled with Hoechst 33342 (blue), cardiomyocytes were labeled with α-actinin (red), and LamR was labeled with an Alexa Fluor 488 secondary antibody (green). Scale bars are 20μm.

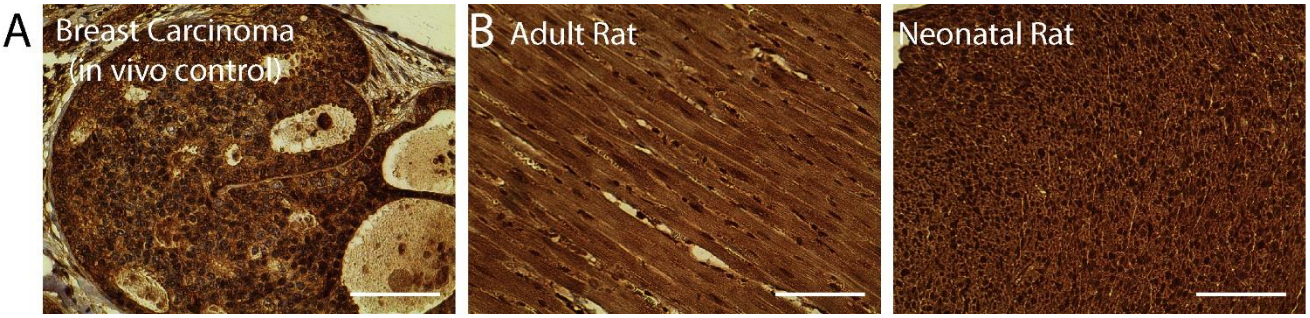

Supplementary Figure 3. LamR is expressed in rat hearts. (**A**) High resolution immunostain of breast carcinoma tissue (*in vivo* positive control). (**B**) High resolution positive immunostains of adult and neonatal rat hearts. Scale bars are 100μm.

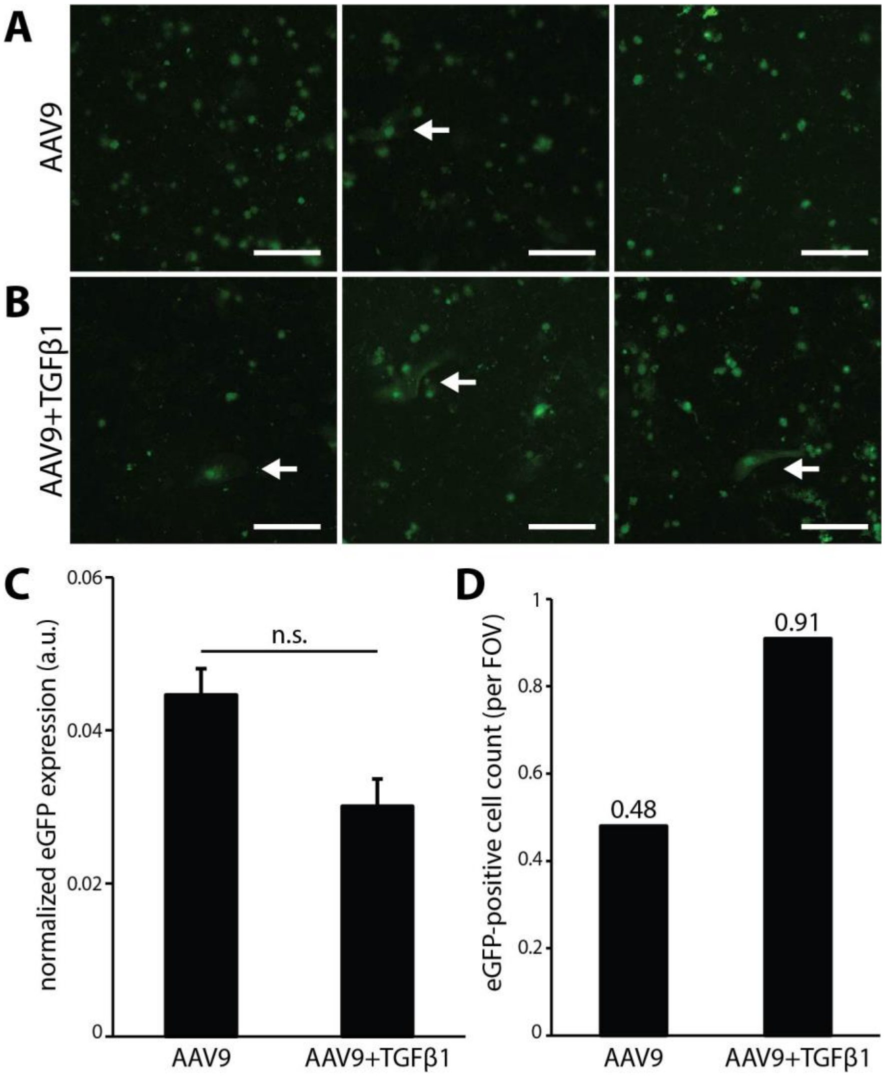

Supplementary Figure 4. AAV9-mediated infection is nominally increased by TGFβ1 treatment. eGFP expression in (**A**) AAV9-infected hiPSC-CMs and (**B**) AAV9-infected hiPSC-CMs pre-treated with 10ng/mL TGFβ1. White arrows point to eGFP expressing cells. (**C**) Pixel threshold-based quantification of eGFP expression in AAV9-infected hiPSC-CMs with and without TGFβ1 treatment showed no significant differences, however (**D**) cell count-based quantification of eGFP expression showed a doubling of eGFP-positive cells per field of view (FOV) with TGFβ1 treatment. Scale bars are 100μm.

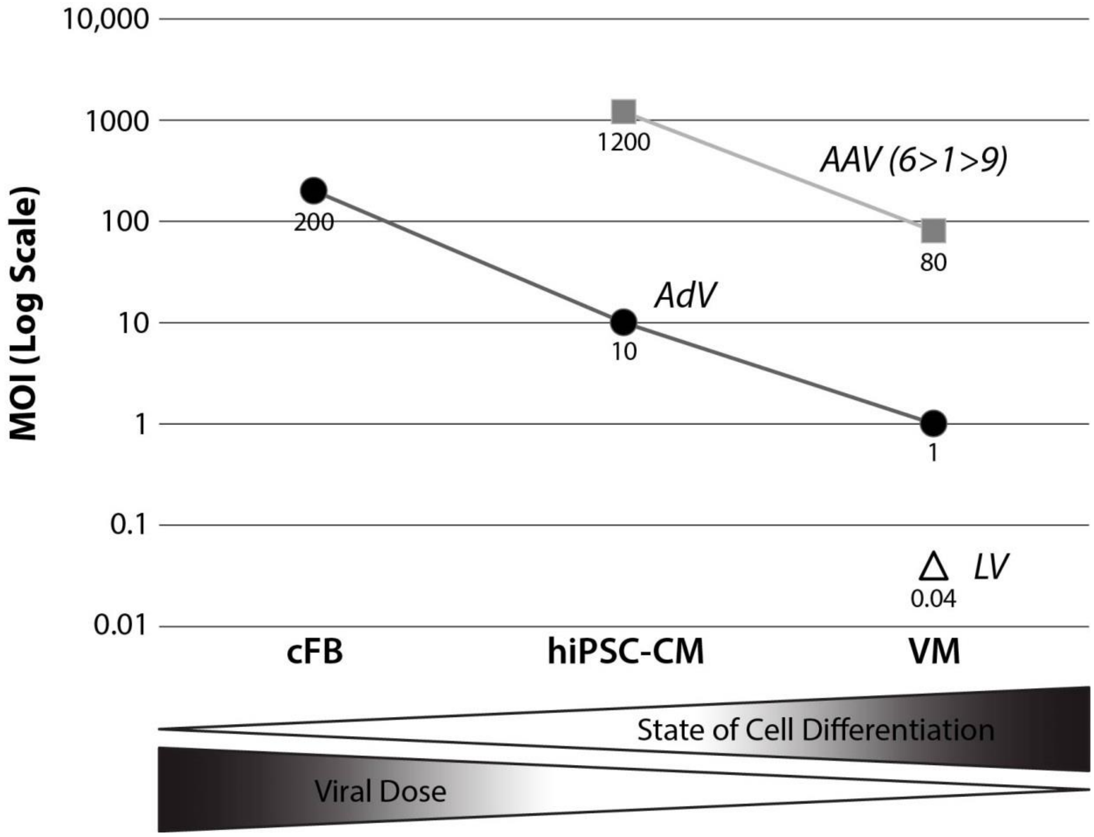

Supplementary Figure 5. Viral dose and cell differentiation state affect viral infection *in vitro*. *In vitro* viral infection is dependent on two factors: the type of viral vector and the state of differentiation of the target cell. cFB (primary cardiac fibroblasts), iPSC-CMs (induced pluripotent stem cell- derived cardiomyocytes), and VMs (primary ventricular myocytes) were infected with AAV, AdV, and/or LV as shown. Optimal viral doses (or MOIs), resulting in >80% transgene expression, are plotted for each cell type and virus (normalized to AdV infection of VMs) on a logarithmic scale. AAVs required the highest MOIs, followed by AdV, whereas LV required an MOI of 1 for optimal infection. In terms of cell type, which in this case is representative of the state of cell differentiation, cFBs required the highest MOIs and VMs required the lowest MOIs for optimal infection.

References

Aikawa, R., Huggins, G.S., and Snyder, R.O. (2002). Cardiomyocyte-specific gene expression following recombinant adeno-associated viral vector transduction. *J Biol Chem* 277(21), 18979-18985. doi: 10.1074/jbc.M201257200.

Ambrosi, C.M., Boyle, P.M., Chen, K., Trayanova, N.A., and Entcheva, E. (2015). Optogenetics-enabled assessment of viral gene and cell therapy for restoration of cardiac excitability. *Sci Rep* 5, 17350. doi: 10.1038/srep17350.

Ambrosi, C.M., and Entcheva, E. (2014). Optogenetic Control of Cardiomyocytes via Viral Delivery. *Methods Mol Biol* 1181, 215-228. doi: 10.1007/978-1-4939-1047-2_19.

Bish, L.T., Morine, K., Sleeper, M.M., Sanmiguel, J., Wu, D., Gao, G., et al. (2008). Adeno-associated virus (AAV) serotype 9 provides global cardiac gene transfer superior to AAV1, AAV6, AAV7, and AAV8 in the mouse and rat. *Hum Gene Ther* 19(12), 1359-1368. doi: 10.1089/hum.2008.123.

Geisler, A., Jungmann, A., Kurreck, J., Poller, W., Katus, H.A., Vetter, R., et al. (2011). microRNA122-regulated transgene expression increases specificity of cardiac gene transfer upon intravenous delivery of AAV9 vectors. *Gene Ther* 18(2), 199-209. doi: 10.1038/gt.2010.141.

Inagaki, K., Fuess, S., Storm, T.A., Gibson, G.A., McTiernan, C.F., Kay, M.A., et al. (2006). Robust systemic transduction with AAV9 vectors in mice: efficient global cardiac gene transfer superior to that of AAV8. *Mol Ther* 14(1), 45-53. doi: 10.1016/j.ymthe.2006.03.014.

Jia, Z., Valiunas, V., Lu, Z., Bien, H., Liu, H., Wang, H.Z., et al. (2011). Stimulating cardiac muscle by light: cardiac optogenetics by cell delivery. *Circ Arrhythm Electrophysiol* 4(5), 753-760. doi: 10.1161/CIRCEP.111.964247.

Kawamoto, S., Shi, Q., Nitta, Y., Miyazaki, J., and Allen, M.D. (2005). Widespread and early myocardial gene expression by adeno-associated virus vector type 6 with a beta-actin hybrid promoter. *Mol Ther* 11(6), 980-985. doi: 10.1016/j.ymthe.2005.02.009.

Klimas, A., Ambrosi, C.M., Yu, J., Williams, J.C., Bien, H., and Entcheva, E. (2016). OptoDyCE as an automated system for high-throughput all-optical dynamic cardiac electrophysiology. *Nat Commun* 7, 11542. doi: 10.1038/ncomms11542.

Klimas, A., Ortiz, G., Boggess, S., Miller, E.W., and Entcheva, E. (2018). Multimodal on-axis platform for all-optical electrophysiology with near-infrared probes in human stem-cell-derived cardiomyocytes. *bioRxiv*. doi: 10.1101/269258.

Kuken, B.N., Aikemu, A.N., Xiang, S.Y., and Wulasihan, M.H. (2015). Effect of SERCA2a overexpression in the pericardium mediated by the AAV1 gene transfer on rapid atrial pacing in rabbits. *Genet Mol Res* 14(4), 13625-13632. doi: 10.4238/2015.October.28.24.

Miyazaki, J., Takaki, S., Araki, K., Tashiro, F., Tominaga, A., Takatsu, K., et al. (1989). Expression vector system based on the chicken beta-actin promoter directs efficient production of interleukin-5. *Gene* 79(2), 269-277.

Muller, O.J., Leuchs, B., Pleger, S.T., Grimm, D., Franz, W.M., Katus, H.A., et al. (2006). Improved cardiac gene transfer by transcriptional and transductional targeting of adeno-associated viral vectors. *Cardiovasc Res* 70(1), 70-78. doi: 10.1016/j.cardiores.2005.12.017.

Pacak, C.A., Mah, C.S., Thattaliyath, B.D., Conlon, T.J., Lewis, M.A., Cloutier, D.E., et al. (2006). Recombinant adeno-associated virus serotype 9 leads to preferential cardiac transduction in vivo. *Circ Res* 99(4), e3-9. doi: 10.1161/01.RES.0000237661.18885.f6.

Palomeque, J., Chemaly, E.R., Colosi, P., Wellman, J.A., Zhou, S., Del Monte, F., et al. (2007). Efficiency of eight different AAV serotypes in transducing rat myocardium in vivo. *Gene Ther* 14(13), 989-997. doi: 10.1038/sj.gt.3302895.

Prasad, K.M., Xu, Y., Yang, Z., Acton, S.T., and French, B.A. (2011). Robust cardiomyocyte-specific gene expression following systemic injection of AAV: in vivo gene delivery follows a Poisson distribution. *Gene Ther* 18(1), 43-52. doi: 10.1038/gt.2010.105.

Pulicherla, N., Shen, S., Yadav, S., Debbink, K., Govindasamy, L., Agbandje-McKenna, M., et al. (2011). Engineering liver-detargeted AAV9 vectors for cardiac and musculoskeletal gene transfer. *Mol Ther* 19(6), 1070-1078. doi: 10.1038/mt.2011.22.

Seiler, M.P., Miller, A.D., Zabner, J., and Halbert, C.L. (2006). Adeno-associated virus types 5 and 6 use distinct receptors for cell entry. *Hum Gene Ther* 17(1), 10-19. doi: 10.1089/hum.2006.17.10.

Wang, Z., Zhu, T., Qiao, C., Zhou, L., Wang, B., Zhang, J., et al. (2005). Adeno-associated virus serotype 8 efficiently delivers genes to muscle and heart. *Nat Biotechnol* 23(3), 321-328. doi: 10.1038/nbt1073.

Wenzel, S., Henning, K., Habbig, A., Forst, S., Schreckenberg, R., Heger, J., et al. (2010). TGF-beta1 improves cardiac performance via up-regulation of laminin receptor 37/67 in adult ventricular cardiomyocytes. *Basic Res Cardiol* 105(5), 621-629. doi: 10.1007/s00395-010-0108-1.

Wu, Z., Miller, E., Agbandje-McKenna, M., and Samulski, R.J. (2006). Alpha2,3 and alpha2,6 N-linked sialic acids facilitate efficient binding and transduction by adeno-associated virus types 1 and 6. *J Virol* 80(18), 9093-9103. doi: 10.1128/JVI.00895-06.

Yang, L., Jiang, J., Drouin, L.M., Agbandje-McKenna, M., Chen, C., Qiao, C., et al. (2009). A myocardium tropic adeno-associated virus (AAV) evolved by DNA shuffling and in vivo selection. *Proc Natl Acad Sci U S A* 106(10), 3946-3951. doi: 10.1073/pnas.0813207106.

Ying, Y., Muller, O.J., Goehringer, C., Leuchs, B., Trepel, M., Katus, H.A., et al. (2010). Heart-targeted adeno-associated viral vectors selected by in vivo biopanning of a random viral display peptide library. *Gene Ther* 17(8), 980-990. doi: 10.1038/gt.2010.44.

Yue, Y., Ghosh, A., Long, C., Bostick, B., Smith, B.F., Kornegay, J.N., et al. (2008). A single intravenous injection of adeno-associated virus serotype-9 leads to whole body skeletal muscle transduction in dogs. *Mol Ther* 16(12), 1944-1952. doi: 10.1038/mt.2008.207.

Zhu, X., McTiernan, C.F., Rajagopalan, N., Shah, H., Fischer, D., Toyoda, Y., et al. (2012). Immunosuppression decreases inflammation and increases AAV6-hSERCA2a-mediated SERCA2a expression. *Hum Gene Ther* 23(7), 722-732. doi: 10.1089/hum.2011.108.

Zincarelli, C., Soltys, S., Rengo, G., and Rabinowitz, J.E. (2008). Analysis of AAV serotypes 1-9 mediated gene expression and tropism in mice after systemic injection. *Mol Ther* 16(6), 1073-1080. doi: 10.1038/mt.2008.76.
